# SHORT syndrome in a two-year-old girl – case report

**DOI:** 10.1186/s13052-017-0362-z

**Published:** 2017-05-04

**Authors:** Maria Klatka, Izabela Rysz, Katarzyna Kozyra, Agnieszka Polak, Witold Kołłątaj

**Affiliations:** 1 Department of Paediatric Endocrinology and Diabetology, Medical University of Lublin, Prof. Antoni Gebala Street 6, PL–20-093 Lublin, Poland; 2 Department of Endocrinology, Medical University of Lublin, Lublin, Poland

**Keywords:** Congenital defects, Short stature, Genetic disease, Rare disease

## Abstract

**Background:**

SHORT syndrome is a rare genetic congenital defects condition. The frequency of the disease still remains unknown.

**Case presentation:**

We report the two-year-four-month old female with SHORT syndrome who present growth retardation and dysmorphic features (triangular-shaped face, prominent forehead, ocular depression, lipodystrophy at the lumbar region and around elbows), consistent with the phenotype described for this syndrome.

The molecular analysis showed the presence of heterozygous variant c.1956dupT (p.Lys653*) in exon 15 of PIK3R1.

**Conclusions:**

The frequency of the disease still remains unknown; solely several dozen cases have been described worldwide.

## Established facts

- In some cases, short stature or slow growth is accompanied by the presence of dysmorphic features
- Among children diagnosed because of short stature, there are patients suffering from very rarely described SHORT syndrome

## Novel insights

- So far there have been found 10 mutations of PIK3R1 leading to appearance of SHORT syndrome symptoms
- Clinical features may vary in different patients
- Appearance of symptoms of SHORT syndrome may depend on the type of genetic defects and DNA nucleotide changes

## Background

SHORT syndrome is an extremely rare genetic condition characterised by multiple congenital defects of internal organs [1–3]. The frequency remains unknown (<1: 1000000 [4]); merely several dozen cases have been described worldwide [5, 6].

It is registered in Orphan diseases database as ORPHA:3163, Phenotype MIM number 269880, Gene/Locus MIM number 171833.

The name of the syndrome is an acronym made up of main clinical characteristics and they are as follows: (S) short stature, (H) hyperextensibility of joints, (O) ocular depression,(R) Rieger anomaly and (T) teething delay [6, 7].

There are different synonyms of SHORT Syndrome that can be found in the literature, among them the most frequently used:

- Growth Retardation-Rieger Anomaly,
- Lipodystrophy, partial, with Rieger anomaly and short stature,
- Reiger Anomaly-Growth Retardation
- Short Stature-Hyperextensibility-Rieger Anomaly-Teething Delay.

The name of the syndrome constitutes the acronym SHORT which stands for typical features found in most of the patients and they are as follows: short stature, hyperextensibility of the joints and/or inguinal hernias, ocular depression (deeply-set eyes), Rieger anomaly and delayed teething [1, 3, 5]. Another feature is intrauterine growth restriction (IUGR). Babies are born with low weight and length. In childhood, slow weight and height gains are observed, and the target height is low. Some characteristic features of the face include triangular-shaped face, prominent forehead, deeply-set eyes, thin or underdeveloped nostrils (hypoplastic nasal alae), thin lips and mouth downturned, a small chin with a dimple, low-set ears, wrinkles [1–3, 5, 6, 8].

Another typical feature of the syndrome is lipodystrophy, namely lack of adipose tissue under the skin mainly on the face, arms and chest. Insufficiency of the adipose tissue makes the blood vessels more visible through the thin and translucent skin. Thus, the patients who suffer from the syndrome look much older than their calendar age shows and the appearance of premature aging is referred to as progeria [9–12].

The patients have underdeveloped anterior chamber of the eye which is called Rieger anomaly. It is the dysgenesis of the iris and cornea with marked hypoplasia of the iris stroma, displacement of the pupil (corectopia) and full-thickness colobomas of the iris (pseudopolycoria). This can be linked to higher ocular pressure and glaucoma development which can lead to vision loss [9]. Most patients require hearing aid implementation because of sensorineural hearing loss. Futhermore, the syndrome also comprises dental abnormalities such as delayed tooth eruption, small teeth (microdontia), decresed number of teeth (hypodontia) and lack of the protective layer on the teeth. Some patients have some urinary tract diseases; nephrocalsinosis is the most common. Delayed bone age, hyperextensibility of the joints, epiphyseal plate thickening as well as clinodactyly (dysmorphic feature of the medial or lateral curvature of the finger or toe, it typically affects the fifth digit).

Carbohydrate metabolism disorder, namely insulin resistance and diabetes mellitus usually occur in the second decade of life [11]. Some patients suffer from congenital heart defects. Intelligence is within normal ranges. Mild cognitive disorders have been described as well as delayed speech development can occur in childhood [1–3, 5, 6].

SHORT syndrome is inherited in an autosomal dominant manner [13]. The proportion of cases caused by de novo mutation is unknown but appears to be significant.

It is caused by mutations of PIK3R1 (phosphoinositide-3-kinase regulatory subunit 1) located on chromosome 5 (5q13.1) that is on the long arm of chromosome 5 with the position 5q13.1. [2, 3]. The proportion of cases caused by de novo mutation is unknown. The human gene PIK3R1 comprises 86102 bp of DNA and contains 16 exons. PIK3R1 includes an instruction on formation of the enzyme subunit which is referred to as PI3K. PI3K signaling is involved in many cellular functions such as growth, proliferation, migration, metabolism, survival and apoptosis. The gene encodes four different protein isoforms CCDS3993 (p85α), CCDS3994 (p55α), CCDS3995(p50α) and CCDS56374 due to alternative splicing. In mammalian tissues, p85α occurs in the brain, liver, adipose tissue, kidneys, spleen; p55α is expressed mainly in the brain and skeletal muscles; the expression of p50α is present in the brain, liver and kidneys [10]. They can be involved in the regulation of hormones and growth factors. They play a vital role in differentiation of preadipocytes to adipocytes and adipocyte function [14, 15]. P85α constitutes a regulatory subunit of PI3K. In cells, it binds to p110α, the catalytic subunit of PI3K and also stabilizes p110α as well as inhibits the basal activity of p110α [11].

The molecular mechanism of the disease can encompass inhibition of the PI3K-AKT-mTOR pathway which is essential in cellular growth and proliferation [10, 16].

The enzyme described above also plays a significant role in metabolism regulation – it is probably responsible for insulin activity. The function of PI3K can be associated with insulin resistance development and consequently diabetes occurrence.

The mutation in several basic elements of the PI3K pathway is linked with some most common conditions, diabetes and cancers, among many others [11, 17, 18].

Autosomal dominant inheritance occurs in the syndrome which means that one copy of the altered gene PIK3R1 is sufficient to develop the disorder. In most cases, de novo mutation is found. It can be transmitted by one of the parents who has got the defected gene [1].

As highlighted above, the syndrome has a tendency for tumourigenesis. The increased PI3K signaling probably favours uncontrolled cellular growth and division which is typical of tumours. Somatic gene mutations PI3K were identified in some uterine cancers and brain cancers, namely gliomas [19–22] but, so far, such carcinogenic mutations have not been diagnosed in patients with SHORT syndrome.

The diagnosis of SHORT syndrome is based on physical findings, X-rays, and molecular genetic testing.

Molecular genetic testing is focused on sequence analysis of PIK3R1 or on use of a multi-gene panel that enables PIK3R1 sequence as well as deletion/duplication analysis.

SHORT syndrome should be differentiated among other syndromes such as Rieger’s, Russell-Silver syndrome, Seckel’s and leprehaunism (Donohue’s syndrome) since they are characterized by numerous common features [6].

Treatment of SHORT syndrome is symptomatic and each patient requires multispecialist care.

Individuals with SHORT syndrome are considered to have a normal life-expectancy.

The paper illustrates the case of a girl suffering from SHORT syndrome with most of the typical features [4–8], caused by the de novo mutation that has never been reported in the literature.

## Aim

The aim was to present a case extremely rare genetic condition characterised by short stature, dysmorphia and congenital defects of internal organs caused by the novel mutation in PIK3R1 -variant c.1956dupT (p.Lys653*) in exon 15 of PIK3R1.

## Method

The following analytical tools were applied to set the proper diagnose:

- anthropometric examination
- biochemical test
- endocrinology tests (insulin resistance test, thyroid function test, HbA_1_c, fructosamine levels)
- eye examination
- impedance audiometry test
- magnetic resonance imaging
- medical history analysis
- molecular genetic testing
- neurology examination
- orthopedic examination
- physical examination
- psychological examination
- psychological tests
- skeleton X-ray
- stomatologic examination
- The ABR test (Auditory Brainstem Response).

## Case presentation

This is a two-year-four-month old female, born by a primigravida. The girl was delivered at 39 week of gestation by the Cesarean section due to placenta failure. The girl was delivered with a general good condition, her Apgar score at 1 min after birth was 9 and she displayed features of intrauterine growth restriction (IUGR). Her birth weight was 1850 g (<3 percentile), height 44.5 cm (<3 percentile), head circumference 31 cm (<3 percentile). From day 7 on, the baby was exclusively breastfed which resulted in gradual weight gain. At 16 day, the baby girl weighed 2018 g and was discharged from hospital with a general good condition.

At the age of three months, the infant was treated at the Paediatric ENT Department for suspected hypoacusis. The Auditory Brainstem Response (ABR) test performed in sleep revealed bilateral severe hearing loss. At follow-up ABR test, sensorineural hearing loss was confirmed. At the age of 4 months, she had a hearing aid placed.

In her infancy, physical developmental delay was diagnosed – her weight and height were substantially below 3 percentile. Her psychomotor development was normal. The girl was able to sit at the age of 8 months and simultaneously she started to stand up and crawl. When she was 14 months old, she began to walk. She started talking when she was a thirteen-month-old. No abnormality was found in the MRI of her central nervous system.

**Fig. 1:**
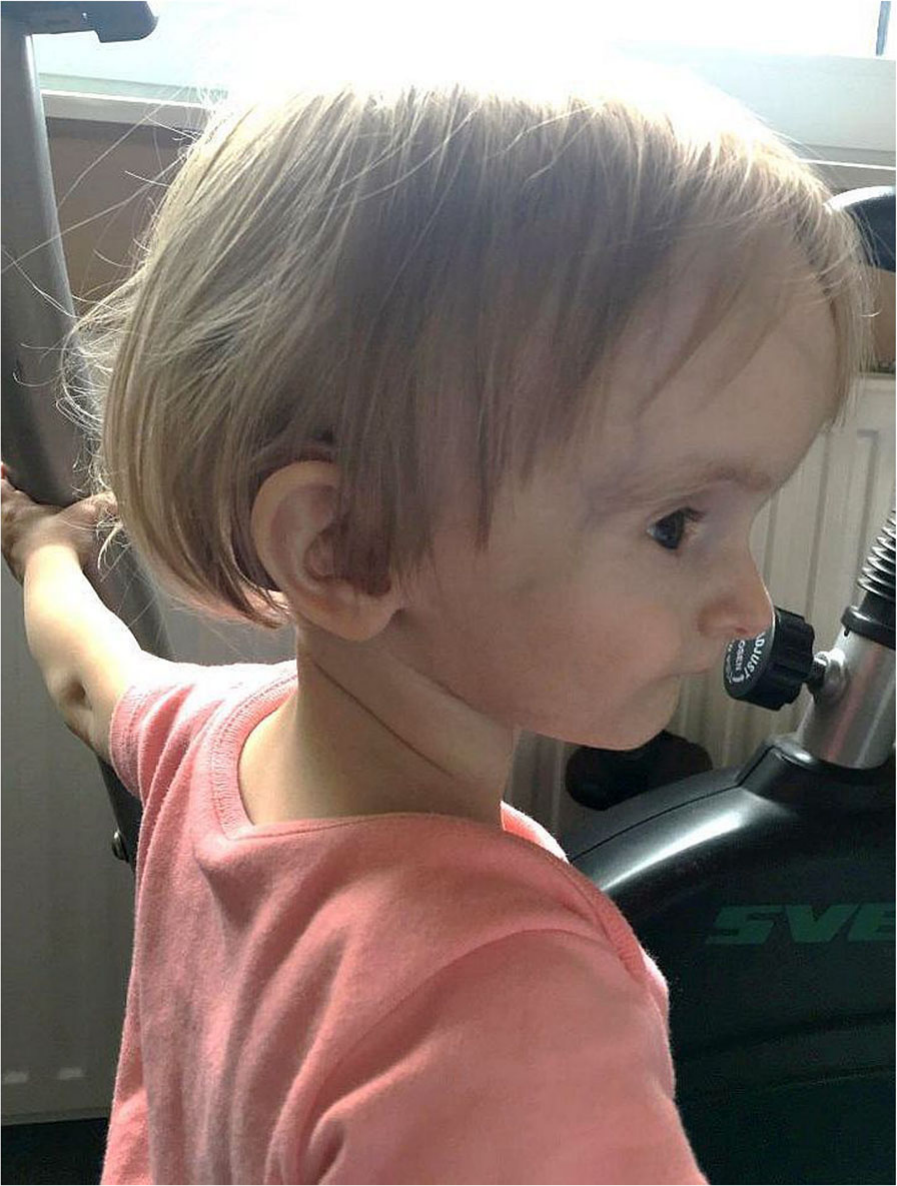
Dysmorphic features (triangular-shaped face with prominent forehead and deeply-set eyes)

**Fig. 2:**
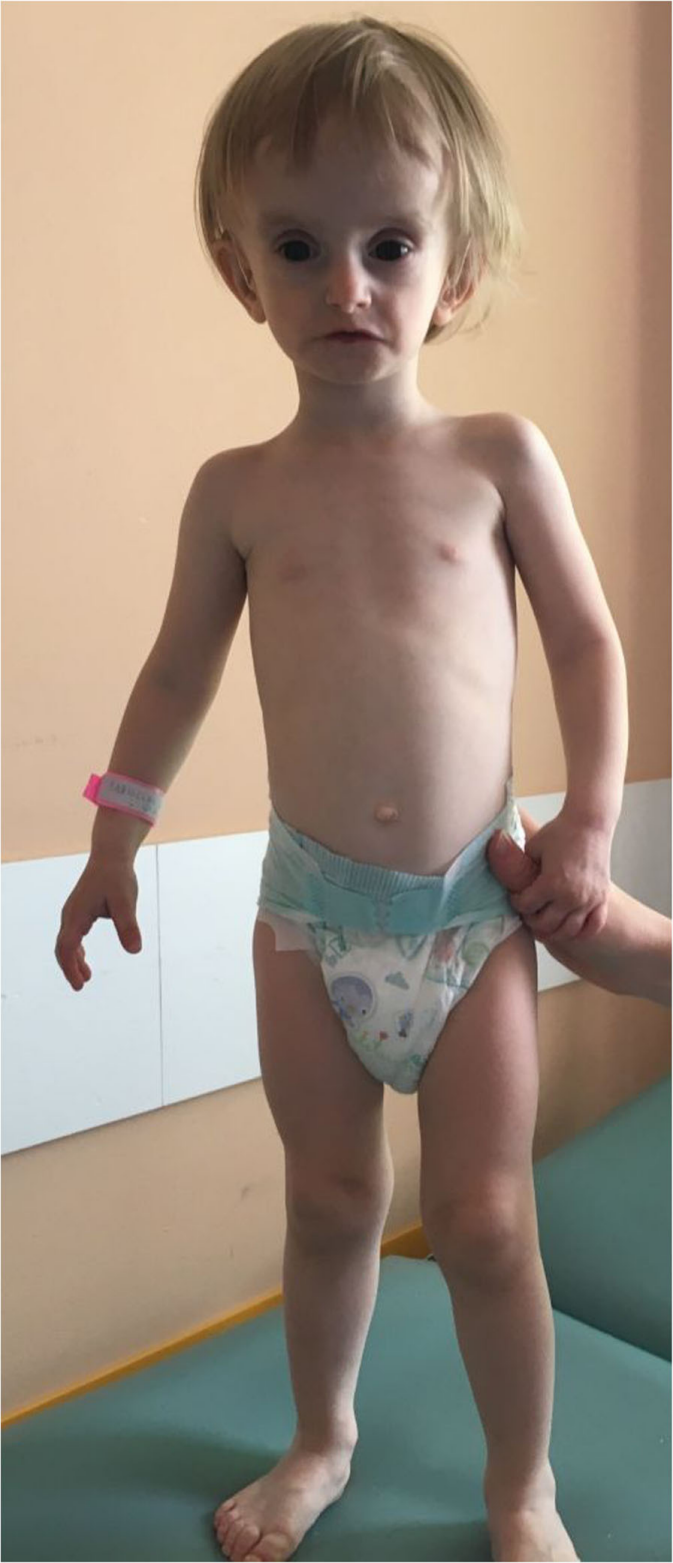
Dysmorphic features and body silhouette - front view

**Fig. 3:**
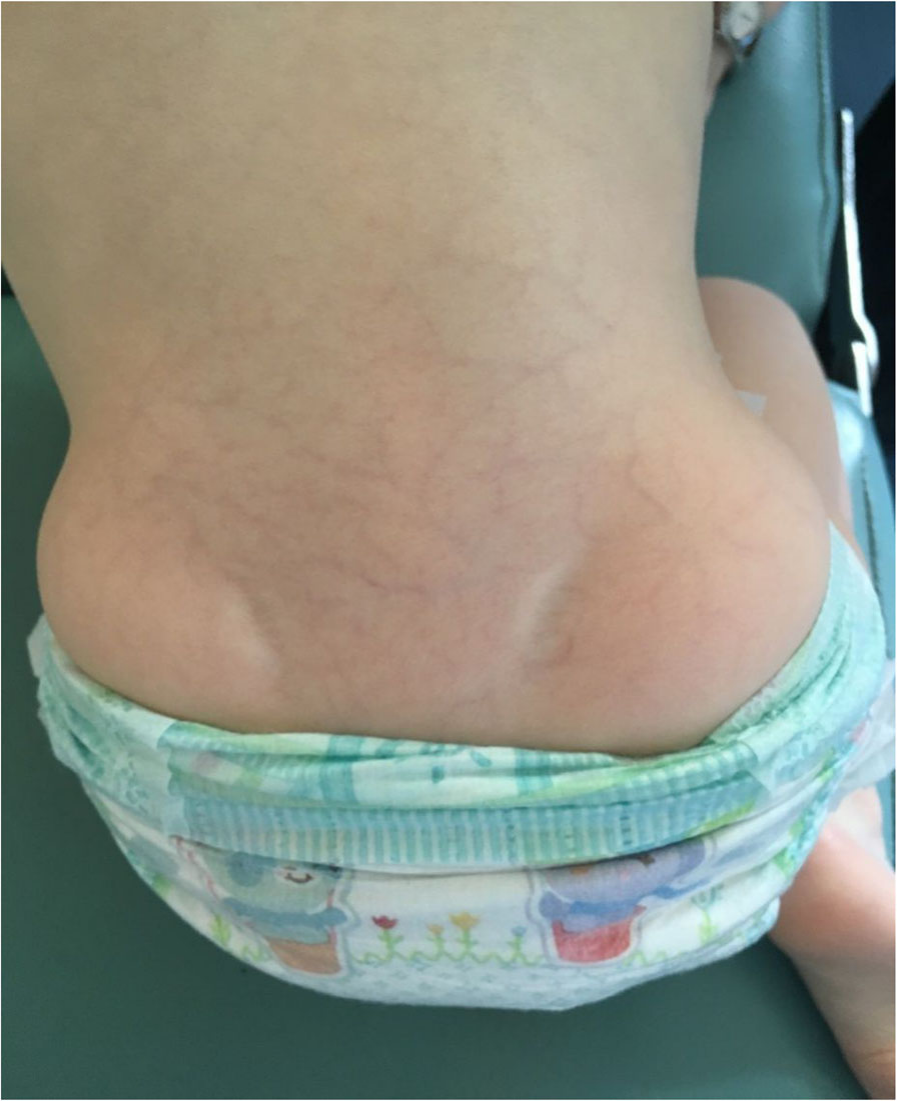
Lipodystrophy at the lumbar region

**Fig. 4:**
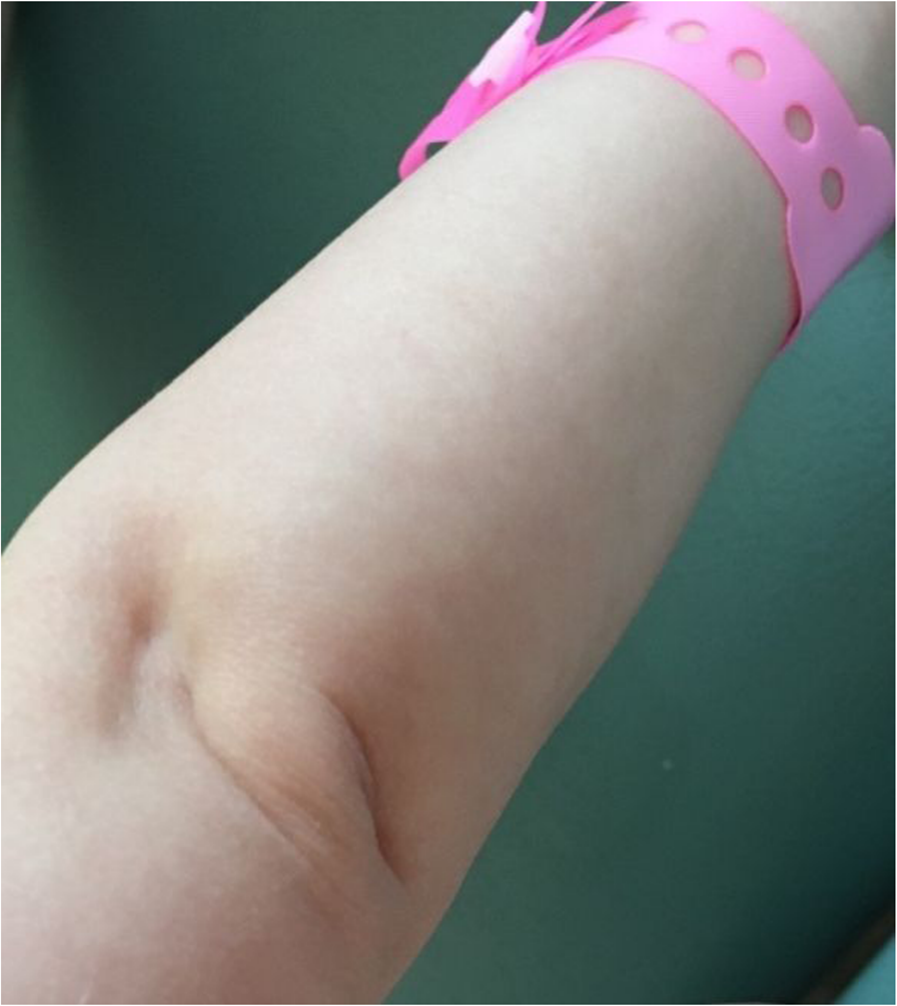
Lipodystrophy around the elbow region

Low weight and height gains, some dysmorphic features (triangular-shaped face, prominent forehead, ocular depression, lipodystrophy at the lumbar region and around elbows (Figs. 1, 2, 3 and 4) led to suspicion of SHORT syndrome.

Our patient presents most common signs and symptoms of SHORT syndrome except Rieger anomaly,insulin intolerance, hyperextensibility of joints and clinodactyly.

The comparison of features typical of SHORT syndrome, with our patient’s features presents Table 1.

**Table 1:** The comparison of features typical of SHORT syndrome, with our patient’s features

Typical features of SHORT syndrome		Our patient’s features
Development	Short stature	+
	Low body weight	+
Phenotypic traits	Triangular face	+
	Prominent forehead	+
	Small chin	+
	Thin or underdeveloped nostrils/ Hypoplastic nasal alae	+
	Large mouth with thin, downturned lips	+
	Low set ears	+
Eyes	Rieger anomaly	-
	Deeply-set eyes	+
	Glaucoma	-
	Cataract	-
	Myopia	-
Teeth	Delayed teething	+
	Small teeth/microdontia	+
	Hypodontia	-
	Lack of protective layer	-
Bones and joints	Delayed bone age	+
	Joint hyperextensibility	-
	Clinodactyly	-
Skin and subcutaneous tissue	Thin and wrinkled skin with well-visible blood vessels	+
	Lipoatrophy	+
Nervous system and cognitive function	Intelligence within normal ranges	+
	Delayed speech development	+
Urinary system	Nephrocalcinosis	-
Endocrine system	Glucose intolerance/ diabetes mellitus	-
	Insulin resistance	-

While preforming molecular genetic testing, we have found a novel mutation in PIK3R1 -variant c.1956dupT (p.Lys653*) in exon 15 of PIK3R1 which is responsible for the symptoms in the patient.

The variant has been confirmed to be de novo mutation (not present in biological parents) that has never been reported in the literature.

At present, the patient is 2 years 4 months old and requires multi-speciality care. Her height and weight remain considerably below 3 percentile, though her psychomotor development is correct.

## Discussion

Clinical criteria for the diagnosis of SHORT syndrome have not been determined yet. However, it is highly feasible that typical facial features and concomitant various congenital anomalies can suggest the disease. The ultimate diagnosis of the syndrome is made on the basis of molecular examination.

The major features described in the SHORT acronym are not universally seen and only half of affected people have 4 or more of the classic features [23].

While comparing our observations to reported cases of SHORT syndrome [1, 11] it can be concluded that the diversity of the clinical picture may largely depend on the type of mutation and the localization of the genetic defect.

The human gene PIK3R1 comprises 86102 bp of DNA and contains 16 exons.

Theoretically, there is the possibility of a large number of genetic defects located in such 16 exons affecting in different way PIK3R1 function in terms of its impact on growth, proliferation, migration, metabolism, survival and apoptosis. May be, some of them are lethal.

So far there have been described only 9 mutations evidently associated with SHORT syndrome (Table 2). The mutation described above it the 10^th^ one.

**Table 2:** Pathogenic PIK3R1 allelic variants in individuals with SHORT syndrome

	DNA nucleotide change	Protein amino acid change	References
1	c.1465G>A	p.Glu489Lys	[11, 24]
2	c.1615_1617delATT	p.Ile539del	[11, 24]
3	c.1892G>A	p.Arg631Gln	[24]
4	c.1906_1907delAA	p.Asn636ProfsTer17	[24]
5	c.1906_1907insC	p.Asn636Thrfs*18	[1]
6	c.1929_1933delTGGCA	p.Asp643Aspfs*8	[8]
7	c.1943dupT	p.Arg649ProfsTer5	[11, 24]
8	c.1945C>T	p.Arg649Trp	[1, 3, 8, 11, 12, 24]
9	c.1971T>G	p.Tyr657Ter	[11, 24]
10	c.1956dupT	p.Lys653*	newly described

It may be drawn a suggestion that c.1956dupT (p.Lys653*) in exon 15 of PIK3R1 rather doesn’t predispose to insulin intolerance but it is yet to be seen in the future. Although insulin resistance is considered as a typical metabolic disorder in patients with SHORT syndrome, there are suggestions that in some cases it occurs in the second decade of life [11]. Insulin resistance has been diagnosed in patients with the following mutations c.1929_1933delTGGCA [8], 1945C>T [11], c.1615_1617delATT [11], c.1465G>A [11], c.1943dupT [11], c.1892G>A [11] although Dyment David A. and co-authors [1] have described an 18 years old individual with c.1945C>T mutation free of insulin resistance problem.

Partial lipodystrophy is considered as universal in SHORT syndrome [24]. It was observed in our patient (Figs. 3 and 4), too. So far its appearance has described in individuals with c.1945C>T [11, 12] as well as c.1615_1617delATT [11], c.1465G>A [11], c.1943dupT [11], c.1892G>A [11] mutations. Usually lipodystrophy as well as insulin resistance have been considered as symptoms appearing together in such individuals.

The girl described above does not present signs of Rieger anomaly. Such anomaly is considered as typical (element of set of symptoms listed in acronym SHORT) but not necessary to diagnose such syndrome.

So far such anomaly has been observed in patients with c.1906_1907insC as well as c.1971T>G and considered as possible in patients with c.1945C>T [1]. Dyment David A. [1] published data concerning 5 individuals with SHORT syndrome and c.1945C>T mutation. 3 of them were evidently free of Rieger anomaly. Bárcena C. and co-authors [8] also described an individual (33-year-old male) with c.1945C>T mutation in an individual without Rieger anomaly.

Our patient doesn’t present symptoms suggesting hyperextensibility of joints or clinodactyly. Such symptoms are common in in people with SHORT syndrome but their appearance is difficult to correlate with the types of genetic defects (for example they may be present [8, 11] or absent [11] in individuals with c.1945C>T mutations).

## Conclusions

1. We found a novel mutation in PIK3R1 -variant c.1956dupT (p.Lys653*) in exon 15.
2. Our patient presents most common signs and symptoms of SHORT syndrome except Rieger anomaly,insulin intolerance, hyperextensibility of joints and clinodactyly.
3. Familiarisation of dysmorphic features is of great significance in the diagnosis of congenital, genetic syndromes and enables early implementation of appropriate management and proper care of the patient.
